# Supplementary material for: Relationship of prostate cancer topography and tumour conspicuity on multiparametric magnetic resonance imaging: a protocol for a systematic review and meta-analysis
Source: BMJ Open. 2022 Jan 5;12(1):e050376. doi: 10.1136/bmjopen-2021-050376 (PMC8734010; doi:10.1136/bmjopen-2021-050376)
Supplement: Author's manuscript [file bmjopen-2021-050376.draft_revisions.pdf]

BMJ Open is committed to open peer review. As part of this commitment we make the peer review history of every article we publish publicly available.

When an article is published we post the peer reviewers' comments and the authors' responses online. We also post the versions of the paper that were used during peer review. These are the versions that the peer review comments apply to.

The versions of the paper that follow are the versions that were submitted during the peer review process. They are not the versions of record or the final published versions. They should not be cited or distributed as the published version of this manuscript.

BMJ Open is an open access journal and the full, final, typeset and author-corrected version of record of the manuscript is available on our site with no access controls, subscription charges or pay-per-view fees (http://bmjopen.bmj.com).

If you have any questions on BMJ Open's open peer review process please email info.bmjopen@bmj.com

BMJ Open

Relationship of prostate cancer topography and tumour conspicuity on multiparametric magnetic resonance imaging: a protocol for a systematic review and meta-analysis

Journal:	*BMJ Open*
Manuscript ID	bmjopen-2021-050376
Article Type:	Protocol
Date Submitted by the Author:	17-Feb-2021
Complete List of Authors:	Satish, Pranav; UCL Medical School, UCL Division of Surgery & Interventional Science<br>Freeman, Alex; University College London Hospitals NHS Foundation Trust, Department of Pathology<br>Kelly, Daniel; Cardiff University, School of Healthcare Sciences<br>Kirkham, Alex; University College London Hospitals NHS Foundation Trust, Department of Radiology<br>Orczyk, Clement; University College London Hospitals NHS Foundation Trust, Department of Urology<br>Simpson, Benjamin Scott; University College London, UCL Cancer Institute<br>Giganti, Francesco; University College London Hospitals NHS Foundation Trust, Department of Radiology<br>Whitaker, Hayley; University College London, Division of Surgery and Interventional Science<br>Emberton, Mark; University College London, Division of Surgery and Interventional Sciences; University College London Hospitals NHS Foundation Trust, Department of Urology<br>Norris, Joseph; University College London, Division of Surgery & Interventional Science
Keywords:	Prostate disease < UROLOGY, Genitourinary imaging < RADIOLOGY & IMAGING, GENITOURINARY MEDICINE

SCHOLARONE™
Manuscripts

Relationship of prostate cancer topography and tumour conspicuity on multiparametric magnetic resonance imaging: a protocol for a systematic review and meta-analysis

Pranav Satish,[1,2] Alex Freeman,[3] Daniel Kelly,[4] Alex Kirkham,[5] Clement Orczyk,[2,6] Benjamin S. Simpson,[7] Francesco Giganti,[2,5] Hayley C. Whitaker,[2] Mark Emberton,[2,6]* Joseph M. Norris,[2,6]*

[1] UCL School of Medicine, University College London, London, UK

[2] UCL Division of Surgery & Interventional Science, University College London, London, UK

[3] Department of Pathology, University College London Hospitals NHS Foundation Trust, London, UK

[4] School of Healthcare Sciences, Cardiff University, Wales, UK

[5] Department of Radiology, University College London Hospitals NHS Foundation Trust, London, UK

[6] Department of Urology, University College London Hospitals NHS Foundation Trust, London, UK

[7] UCL Cancer Institute, University College London, London, UK

** joint-senior authors*

For peer review only

ARTICLE TYPE: Systematic review protocol

WORD COUNT: 1,800 words

KEY WORDS: conspicuity; multiparametric MRI; prostate cancer; systematic review; tumour location

CORRESPONDING AUTHOR: Pranav Satish. UCL Medical School, 74 Huntley Street, Bloomsbury, London, UK, WC1E 6DE. pranav.satish.18@ucl.ac.uk.

CONFLICTS OF INTEREST: JMN receives funding from the MRC. BSS receives funding from the Rosetrees Trust. HCW receives funding from PCUK, the Urology Foundation and the Rosetrees Trust. AK, AF and ME have stock interest in Nuada Medical. ME receives funding from

NIHR-i4i, MRC, Cancer Research UK, Jon Moulton Charitable Foundation, Sonacare Inc., Trod Medical, Cancer Vaccine Institute and Sophiris Biocorp for trials in prostate cancer. Emberton acts as a consultant, and/or trainer and proctor to Sonatherm Inc., Angiodynamics Inc. and Exact imaging Inc.

ETHICS: Not required.

FUNDING: Norris is funded by the MRC (MR/S00680X/1). Francesco Giganti is funded by the UCL Graduate Research Scholarship and the Brahm PhD scholarship in memory of Chris Adams.

For peer review only

ABSTRACT

Introduction:

Multiparametric magnetic resonance imaging (mpMRI) has improved the triage of men with suspected prostate cancer, through precision pre-biopsy identification of clinically significant disease. Multiple important characteristics, including tumour grade and size have been shown to affect the conspicuity of prostate cancer on mpMRI, however, the association of tumour location and mpMRI visibility is an under-explored facet of this field. The objective of this systematic review and meta-analysis is to collate the extant evidence regarding the effect of prostate cancer zonal location on mpMRI conspicuity, for the first time. This review will help clarify mechanisms that underpin whether or not a tumour is visible, and the prognostic implications of our findings.

Methods and analysis:

The MEDLINE, PubMed, Embase and Cochrane databases will be systematically searched for relevant studies. Eligible studies will be full-text English-language articles that examine the effect of zonal location on mpMRI conspicuity. Two reviewers will perform study selection, data extraction and quality assessment. A third reviewer will be involved if consensus is not achieved. The Preferred Reporting Items for Systematic Reviews and Meta-Analyses (PRISMA) guidelines will inform the methodology and reporting of the review. Study bias will be assessed using a modified Newcastle-Ottawa scale. A thematic approach will be used to synthesise key location-based factors associated with mpMRI conspicuity. If there are enough eligible studies, a meta-analysis will be conducted to form a pooled value of the sensitivity and specificity of mpMRI at different tumour locations.

Ethics and dissemination

Ethical approval is not required as it is a protocol for a systematic review. Findings will be disseminated through peer-reviewed publications and conference presentations.

PROSPERO registration number

CRD42021228087

Strengths and limitations of this study

- This systematic review protocol follows the Preferred Reporting Items for Systematic Review and Meta-Analysis Protocols guidelines.
- This review addresses a clinically important gap in the literature with a detailed assessment of the crucial questions surrounding invisible mpMRI lesions
- Due to the limited evidence on the topic the included evidence will be retrospective cohort studies, limiting the strength of conclusions drawn for the thematic synthesis and meta-analysis
- The degree of heterogeneity may limit the generalisability of the results

BACKGROUND

Multiparametric magnetic resonance imaging (mpMRI) has improved the triage of men with suspected prostate cancer, through precision pre-biopsy identification of significant disease. Nevertheless, anywhere from 7% to 55% of prostate cancer lesions may be overlooked by mpMRI[1,2]. As a result, identifying factors that influence visibility of clinically significant cancer has been the subject of extensive research over the past few years[1,3,4]. Whilst multiple important characteristics, including tumour grade and size have been shown to affect conspicuity on mpMRI[4,5], tumour location and association with mpMRI visibility remains an under-explored facet of this field.

Although the link between tumour location and conspicuity on mpMRI has not yet been fully elucidated, location does appear to be associated with clinical risk. Transitional zone (TZ) tumours have been associated with lower pathological grade disease[6] and lower rates of biochemical failure following radical prostatectomy[7]. There is also increasing evidence to suggest mpMRI-visible lesions may associate with more aggressive clinical features[8]. Given this growing body of evidence linking risk,

location and conspicuity on mpMRI, the need to systematically appraise the association of these features is evident. Therefore, the objective of this systematic review and meta-analysis is to collate the extant evidence regarding the effect of prostate cancer zonal location on mpMRI conspicuity, for the first time. This review will help clarify mechanisms that underpin whether or not a tumour is visible, and the prognostic implications of our findings.

METHODS AND ANALYSIS

This systematic review protocol has been written in line with the Preferred Reporting Items for Systematic Reviews and Meta-Analyses Protocol (PRISMA) 2015 checklist[9]. The review was prospectively registered with the PROSPERO review database (CRD42021228087). A systematic review of the PubMed, MEDLINE, Embase and Cochrane databases will be conducted, with the search strategy containing medical subject heading (MeSH) terms for maximum yield of the relevant literature. The search will include MeSH terms for 'prostate cancer' and 'mpMRI,' combined via Boolean operators with synonyms for 'conspicuity' and 'location.' Articles identified by the search strategy will be uploaded to Rayyan[10], a systematic review tool to facilitate the screening process. The included articles' reference section will be screened for further relevant literature not picked up by the initial search strategy.

STUDY SELECTION AND DATA EXTRACTION

The screening process will be conducted by two independent reviewers, removing irrelevant articles based on titles and abstracts. Pertinent studies will be retrieved, and their full text assessed to see if they meet the inclusion criteria. Any dispute between reviewers over the relevance of a study will be discussed until concordance is achieved, or a third reviewer will be consulted in case of persistent discordance. The reasons for exclusion will be documented and detailed in the PRISMA flow diagram. Before starting screening, to minimise inter-reviewer bias, we will conduct calibration exercises to maintain consistency between the two reviewers.

INCLUSION CRITERIA

Studies must investigate one or more aspects of the location of prostate cancer in relation to the visibility on mpMRI. Zonal location may be defined as traditional McNeal zones[11], biopsy-derived data such as Barzell zones[12], or data acquired via the Ginsburg protocol[13], or any other recognised approach of describing prostate topography. More general definitions of location such as anterior, posterior, base, midgland and apex will also be included. Visibility on mpMRI must be measured by any version of the Prostate Imaging-Reporting and Data System (PI-RADS)[14], Likert scoring system[15], which is widely used in the UK, or centre-specific scoring system.

EXCLUSION CRITERIA

Conference abstracts, expert opinions, correspondence articles and case reports will be excluded. Studies that do not correlate zonal location and mpMRI conspicuity will be excluded, as will studies not in the English-language.

DATA EXTRACTION

Reviewers will then independently extract the following information to collate into a separate data sheet: year of publication, authors, study design, patient population, number and age of study participants, mpMRI scoring scheme used (PI-RADS or Likert), definition of clinically significant disease, definition of tumour visibility, sample processing approach, zone(s) assessed and the differential quantification of tumour location (sensitivity, specificity, positive predictive value, negative predictive value and AUC). Table 1 summarises data extraction items.

Item	Data Extracted	Data type
1	Author	Study characteristic
2	Publication year	Study characteristic
3	Study design	Study characteristic
4	Patient Population	Demographic

For peer review only

5	Number of patients	Demographic
6	Patient age	Demographic
7	Prostate specific antigen (PSA)	Demographic
8	mpMRI protocol	Methodology
9	mpMRI scoring scheme	Methodology
10	Method of pathological correlation	Methodology
11	Definition of clinically significant cancer	Methodology
12	Definition of lesion visibility	Methodology
13	Zones assessed	Outcome
14	Differential quantification of conspicuity	Outcome

Table 1: Data extraction items

For peer review

ENDPOINTS

The primary endpoint will be the differential tumour visibility on mpMRI, as stratified by zonal location. Secondary endpoints will include explanatory links between location and mpMRI conspicuity, as well as the potential clinical implications. As study methodology may impact the determination of the zone, we will include these variables in a moderator analysis (see Meta-analysis section).

ASSESSMENT OF BIAS

A modified Newcastle-Ottawa scale to assess bias in non-randomised studies will be calculated across included studies, as previously described[16,17,18]. For cohort studies, the study design most relevant to this review, the system is divided into three core elements: selection, comparability and outcome. Within each section, questions evaluate the quality of the research methodology, at the individual study-level. Both reviewers will independently calculate the score for each study, and any disagreement will be settled by consensus or the involvement of a third reviewer if consensus cannot be achieved. The

outcome of the bias and quality assessment will inform the thematic synthesis by providing an assessment of the reliability and applicability of the available evidence. If studies are deemed to be of excessively low quality (or high bias), then these may be excluded at the discretion and consensus of both reviewers. Or, if included, will be accompanied by qualifying commentary in the discussion. As this review is focused on the location of tumour associated conspicuity on mpMRI (as opposed to treatment outcome), we will modify non-applicable sections of the scoring scheme to more closely reflect the nature of the evidence base.

META-ANALYSIS

As previously described[18], if there are a sufficient number of studies available that analyse location, using sufficiently comparable methodologies as agreed by both reviewers, then we will conduct a meta-analysis. For each topological location reported across studies, the total number of lesions with prostate cancer (m_i), the total number of patients without prostate cancer (n_i) the true positives (y_i, mpMRI visible lesions with confirmed cancer), false positives (z_i, mpMRI visible lesions which are histologically negative), true negatives (m_i-y_i, mpMRI invisible and histologically negative) and false negative (n_i-z_i, mpMRI invisible and present on histological examination) will be recorded. In the case of studies which do not give sufficient detail to derive these values but do list sensitivity or specificity and total patient numbers, these values will be reverse engineered, for example in the case of a study which reports mpMRI sensitivity for a TZ lesion at 0.788 in a group of 60, the true positives can be calculated as $y_i = m_i p_i$ rounded to the nearest integer *or TP = 60 x 0.788 = 47.28* (47.3 true positives). If reverse engineering is not possible, the corresponding author of the included paper will be contacted to try and extract the relevant data. The respective sensitivities and specificities of mpMRI for tumours within differing zonal locations will then be compared. The distribution of untransformed, logit and double arcsine transformed sensitivities and specificities will be compared. Whichever distributions resemble a normal distribution (assessed using density plots and Shapiro-Wilk tests) will be used for further analysis.

The model fitted will be determined based on interstudy variation (measured via I^2); if significant, a random-effect model will be fitted, or a fixed-effect model if not. After fitting a model to all relevant studies, leave-one-out (LOO) analyses and accompanying diagnostic plots will be used to identify influential studies, including externally studentised residuals, difference in fits values, Cook's distances, covariance ratios, LOO estimates of the amount of heterogeneity, LOO values of the test statistics for heterogeneity, hat values and weights. Studies with a statistically significant influence on the fitted model will be removed as outliers and the model refitted. These outliers will be examined for potential confounding variables such as study methodology or poor interobserver agreement for mpMRI scans. For each tumour location individual models will first be fit and the pooled sensitivities, specificities, NPV and TPV will be reported. Finally, using tumour location as a subgroup, a bivariate model will be fit to the data and any differences in sensitivity and specificity will be compared using SROC curves and tested using the Chi-squared likelihood-ratio test. If appropriate, moderator analysis will also be performed between factors, which may influence the outcome, including, but not limited to, study methodology, study size, year of publication, cohort type, relevant methodology (such as radical prostatectomy vs. biopsy studies) or visibility definition. Analytics will be performed as outlined by Wang[19]. If there is insufficient data to conduct a meta-analysis, only thematic synthesis will be performed.

DISCUSSION

Following Level 1 evidence support of mpMRI-directed prostate cancer diagnosis[1,20,21,22] and implementation into national and international clinical guidelines[23], mpMRI has moved to the forefront of prostate cancer diagnosis. Whilst mpMRI provides refinement of the traditional transrectal ultrasound (TRUS)-guided biopsy approach[1], not all significant prostate cancers are visible on mpMRI, and it is pertinent for us now to explore the reasons that underpin this non-visibility. Through this systematic review, we will identify the correlation between zonal location and conspicuity, to enhance our understanding of the factors that influence prostate cancer visibility on mpMRI.

Unusual radiological manifestations of prostate cancer are not uncommon due to nuanced histopathological landscape in each zone, rendering MRI signal generation highly varied. In the peripheral zone, several radiological 'hiding places' exist, such as very distal apical tumours, subcapsular crescentic tumours, tumours mimicking the posterior midline and infiltrative peripheral zone tumours[24]. In the anterior prostate, the presence of benign prostatic hyperplasia (BPH) in the TZ has been known to generate false positive results, due to difficulty discriminating prostate cancer from BPH nodules[25]. Furthermore, MRI signal properties of TZ tumours and normal TZ tissue can be similar[26], further complicating this issue. In the apex of the prostate, the apical capsule is less conspicuous on imaging, and periprostatic fat is usually sparse or completely absent in the apex, which may make the tumour less visible[27]. Nevertheless, one study showed high detection rates for this type of tumour[28]. Given the many, sometimes contradictory, stances surrounding zonal location and conspicuity in the literature, the planned systematic review will discuss these features, amongst others, in detail.

In summary, this systematic review and meta-analysis will combine the extant evidence in this emerging field, for the first time. Collation and analysis of these data will enrich our understanding of the effect of zonal location on the conspicuity of prostate cancer on mpMRI. Additionally, this process will help reveal the potential clinical role that these effects play in both diagnosis and treatment, thereby aiding identification of important avenues for future research.

TRIAL STATUS

- Preliminary searches: started.
- Piloting of the study selection process: started.
- Formal screening: started
- Data extraction: not started.
- Risk of bias assessment: not started.
- Data analysis: not started.

Draft of search strategy for MEDLINE, EMBASE, PubMed and Cochrane databases

(Prostate cancer OR prostate tumo*r OR prostate lesion)

AND (region* OR position* OR location* OR zone OR zonal location OR midgland OR base OR apex OR AFMS OR anterior OR posterior)

AND (mpMRI OR multiparametric MRI OR multiparametric MR imaging OR mp-mri)

AND (conspicuity OR visibility OR detect* OR visuali* OR identif*)

ETHICS AND DISSEMINATION

Due to the nature of the study, there are no relevant ethical concerns and informed consent will not be required. The protocol and systematic review will be disseminated via a peer-reviewed journal.

REFERENCES

1. Ahmed HU, El-Shater Bosaily A, Brown LC, Gabe R, Kaplan R, Parmar MK, et al. Diagnostic accuracy of multi-parametric MRI and TRUS biopsy in prostate cancer (PROMIS): a paired validating confirmatory study. The Lancet [Internet]. 2017 Feb;389(10071):815–22. Available from: https://www.thelancet.com/journals/lancet/article/PIIS0140-6736(16)32401-1/fulltext

2. Johnson DC, Raman SS, Mirak SA, Kwan L, Bajgiran AM, Hsu W, et al. Detection of Individual Prostate Cancer Foci via Multiparametric Magnetic Resonance Imaging. European Urology. 2019 May;75(5):712–20.

3. Norris JM, Carmona Echeverria LM, Bott SRJ, Brown LC, Burns-Cox N, Dudderidge T, et al. What Type of Prostate Cancer Is Systematically Overlooked by Multiparametric Magnetic Resonance Imaging? An Analysis from the PROMIS Cohort. European Urology. 2020 Aug;78(2):163–70.

4. Huebner NA, Korn S, Resch I, Grubmüller B, Gross T, Gale R, et al. Visibility of significant prostate cancer on multiparametric magnetic resonance imaging (MRI)—do we still need contrast media? European Radiology. 2020 Dec 2;

5. Bratan F, Niaf E, Melodelima C, Chesnais AL, Souchon R, Mège-Lechevallier F, et al. Influence of imaging and histological factors on prostate cancer detection and localisation on multiparametric MRI: a prospective study. European Radiology. 2013 Mar 15;23(7):2019–29.

6. Lee JJ, Thomas I-C, Nolley R, Ferrari M, Brooks JD, Leppert JT. Biologic differences between peripheral and transition zone prostate cancer. The Prostate. 2014 Oct 18;75(2):183–90.

7. Shin N, Park SY. Postoperative Biochemical Failure in Patients With PI-RADS Category 4 or 5 Prostate Cancers: Risk Stratification According to Zonal Location of an Index Lesion. American Journal of Roentgenology. 2020 Oct;215(4):913–9.

8. Norris JM, Simpson BS, Freeman A, Kirkham A, Whitaker HC, Emberton M. Conspicuity of prostate cancer on multiparametric magnetic resonance imaging: A cross-disciplinary translational hypothesis. The FASEB Journal. 2020 Sep 13;34(11):14150–9.

9. Shamseer L, Moher D, Clarke M, Ghersi D, Liberati A, Petticrew M, et al. Preferred reporting items for systematic review and meta-analysis protocols (PRISMA-P) 2015: elaboration and explanation. BMJ. 2015 Jan 2;349(jan02 1):g7647–g7647.

10. Ouzzani M, Hammady H, Fedorowicz Z, Elmagarmid A. Rayyan—a web and mobile app for systematic reviews. Systematic Reviews [Internet]. 2016 Dec;5(1). Available from: https://systematicreviewsjournal.biomedcentral.com/articles/10.1186/s13643-016-0384-4

For peer review only

11. McNeal JE. The zonal anatomy of the prostate. The Prostate. 1981;2(1):35–49.

12. Valerio M, Anele C, Charman SC, van der Meulen J, Freeman A, Jameson C, et al. Transperineal template prostate-mapping biopsies: an evaluation of different protocols in the detection of clinically significant prostate cancer. BJU International. 2015 Sep 25;118(3):384–90.

13. Hansen N, Patruno G, Wadhwa K, Gaziev G, Miano R, Barrett T, et al. Magnetic Resonance and Ultrasound Image Fusion Supported Transperineal Prostate Biopsy Using the Ginsburg Protocol: Technique, Learning Points, and Biopsy Results. European Urology. 2016 Aug;70(2):332–40.

14. Turkbey B, Rosenkrantz AB, Haider MA, Padhani AR, Villeirs G, Macura KJ, et al. Prostate Imaging Reporting and Data System Version 2.1: 2019 Update of Prostate Imaging Reporting and Data System Version 2. European Urology [Internet]. 2019 Sep 1;76(3):340–351. Available from: https://www.europeanurology.com/article/S0302-2838(19)30180-0/fulltext

15. Dickinson L, Ahmed HU, Allen C, Barentsz JO, Carey B, Futterer JJ, et al. Scoring systems used for the interpretation and reporting of multiparametric MRI for prostate cancer detection, localization, and characterization: could standardization lead to improved utilization of imaging within the diagnostic pathway? Journal of Magnetic Resonance Imaging. 2012 May 7;37(1):48–58.

16. Norris JM, Simpson BS, Ball R, Freeman A, Kirkham A, Parry MA, et al. A Modified Newcastle-Ottawa Scale for Assessment of Study Quality in Genetic Urological Research. European Urology. 2020 Dec;

For peer review only

17. Norris JM, Simpson BS, Parry MA, Allen C, Ball R, Freeman A, et al. Genetic Landscape of Prostate Cancer Conspicuity on Multiparametric Magnetic Resonance Imaging: A Systematic Review and Bioinformatic Analysis. European Urology Open Science. 2020 Jul;20:37–47.

18. Norris JM, Carmona Echeverria LM, Simpson BS, Ball R, Freeman A, Kelly D, et al. Histopathological features of prostate cancer conspicuity on multiparametric MRI: protocol for a systematic review and meta-analysis. BMJ Open. 2020 Oct;10(10):e039735.

19. Wang N. How to conduct a meta-analysis of proportions in R: a comprehensive tutorial, 2018.

20. Kasivisvanathan V, Rannikko AS, Borghi M, Panebianco V, Mynderse LA, Vaarala MH, et al. MRI-Targeted or Standard Biopsy for Prostate-Cancer Diagnosis. New England Journal of Medicine. 2018 May 10;378(19):1767–77.

21. Simmons LAM, Kanthabalan A, Arya M, Briggs T, Barratt D, Charman SC, et al. The PICTURE study: diagnostic accuracy of multiparametric MRI in men requiring a repeat prostate biopsy. British Journal of Cancer. 2017 Mar 28;116(9):1159–65.

22. Rouvière O, Puech P, Renard-Penna R, Claudon M, Roy C, Mège-Lechevallier F, et al. Use of prostate systematic and targeted biopsy on the basis of multiparametric MRI in biopsy-naive patients (MRI-FIRST): a prospective, multicentre, paired diagnostic study. The Lancet Oncology [Internet]. 2019 Jan [cited 2019 Nov 13];20(1):100–9. Available from: https://www.thelancet.com/journals/lanonc/article/PIIS1470-2045(18)30569-2/fulltext

For peer review only

23. Wise J. NICE recommends MRI for suspected prostate cancer to reduce biopsies. BMJ. 2018 Dec 13;k5290.

24. Rosenkrantz AB, Verma S, Turkbey B. Prostate Cancer: Top Places Where Tumors Hide on Multiparametric MRI. American Journal of Roentgenology. 2015 Apr;204(4):W449–56.

25. Hoeks CMA, Hambrock T, Yakar D, Hulsbergen–van de Kaa CA, Feuth T, Witjes JA, et al. Transition Zone Prostate Cancer: Detection and Localization with 3-T Multiparametric MR Imaging. Radiology. 2013 Jan;266(1):207–17.

26. Akin O, Sala E, Moskowitz CS, Kuroiwa K, Ishill NM, Pucar D, et al. Transition Zone Prostate Cancers: Features, Detection, Localization, and Staging at Endorectal MR Imaging. Radiology. 2006 Jun;239(3):784–92.

27. Tan N, Margolis DJ, Lu DY, King KG, Huang J, Reiter RE, et al. Characteristics of Detected and Missed Prostate Cancer Foci on 3-T Multiparametric MRI Using an Endorectal Coil Correlated With Whole-Mount Thin-Section Histopathology. American Journal of Roentgenology. 2015 Jul;205(1):W87–92.

28. Nix JW, Turkbey B, Hoang A, Volkin D, Yerram N, Chua C, et al. Very distal apical prostate tumours: identification on multiparametric MRI at 3 Tesla. BJU International. 2012 Oct 4;110(11b):E694–700.

FOOTNOTES

Twitter: @PranavSatish7, @MrJosephNorris, @b3nsimp, @HayleyWhitaker, @EmbertonMark, @profdkelly, @giga_fra

Contributors: The authors' contribution includes, but is not limited to, the following: PS and JMN drafted the manuscript and created the study concept. BS provided the meta-analysis section. FG, AF, DK, AK, CO, HCW and ME provided supervision and guidance. All authors reviewed and approved the manuscript in its current form.

Funding: JMN is funded by the Medical Research Council (MRC) on an MRC Clinical Research Training Fellowship (Ref: MR/S00680X/1). BSS receives funding from the Rosetrees Foundation. Francesco Giganti is funded by the UCL Graduate Research Scholarship and the Brahm PhD scholarship in memory of Chris Adams.

Competing interests: JMN receives funding from the MRC. BSS receives funding from the Rosetrees Trust. HCW receives funding from PCUK, the Urology Foundation and the Rosetrees Trust. AK, AF and ME have stock interest in Nuada Medical. ME receives funding from NIHR-i4i, MRC, Cancer Research UK, Jon Moulton Charitable Foundation, Sonacare Inc., Trod Medical, Cancer Vaccine Institute and Sophiris Biocorp for trials in prostate cancer. Emberton acts as a consultant, and/or trainer and proctor to Sonatherm Inc., Angiodynamics Inc. and Exact imaging Inc.

Patient and public involvement; Patients and/or the public were not involved in the design, conduct, reporting or dissemination plans of this research.

Patient consent for publication: Not required.

Provenance and peer review: Not commissioned; externally peer reviewed.

For peer review only

For peer review only

Reporting checklist for protocol of a systematic review and meta analysis.

Based on the PRISMA-P guidelines.

Instructions to authors

Complete this checklist by entering the page numbers from your manuscript where readers will find each of the items listed below.

Your article may not currently address all the items on the checklist. Please modify your text to include the missing information. If you are certain that an item does not apply, please write "n/a" and provide a short explanation.

Upload your completed checklist as an extra file when you submit to a journal.

In your methods section, say that you used the PRISMA-Preporting guidelines, and cite them as:

Moher D, Shamseer L, Clarke M, Ghersi D, Liberati A, Petticrew M, Shekelle P, Stewart LA. Preferred Reporting Items for Systematic Review and Meta-Analysis Protocols (PRISMA-P) 2015 statement. Syst Rev. 2015;4(1):1.

		Reporting Item	Page Number
**Title**			
Identification	#1a	Identify the report as a protocol of a systematic review	7
Update	#1b	If the protocol is for an update of a previous systematic review, identify as such	n/a

For peer review only

Registration

	#2	If registered, provide the name of the registry (such as PROSPERO) and registration number	5

Authors

Contact	#3a	Provide name, institutional affiliation, e-mail address of all protocol authors; provide physical mailing address of corresponding author	3
Contribution	#3b	Describe contributions of protocol authors and identify the guarantor of the review	18

Amendments

	#4	If the protocol represents an amendment of a previously completed or published protocol, identify as such and list changes; otherwise, state plan for documenting important protocol amendments	n/a

Support

Sources	#5a	Indicate sources of financial or other support for the review	3
Sponsor	#5b	Provide name for the review funder and / or sponsor	n/a
Role of sponsor or funder	#5c	Describe roles of funder(s), sponsor(s), and / or institution(s), if any, in developing the protocol	n/a

Introduction

For peer review only

Rationale	#6	Describe the rationale for the review in the context of what is already known	6
Objectives	#7	Provide an explicit statement of the question(s) the review will address with reference to participants, interventions, comparators, and outcomes (PICO)	7
**Methods**			
Eligibility criteria	#8	Specify the study characteristics (such as PICO, study design, setting, time frame) and report characteristics (such as years considered, language, publication status) to be used as criteria for eligibility for the review	8
Information sources	#9	Describe all intended information sources (such as electronic databases, contact with study authors, trial registers or other grey literature sources) with planned dates of coverage	7
Search strategy	#10	Present draft of search strategy to be used for at least one electronic database, including planned limits, such that it could be repeated	13
Study records - data management	#11a	Describe the mechanism(s) that will be used to manage records and data throughout the review	7
Study records - selection process	#11b	State the process that will be used for selecting studies (such as two independent reviewers) through each phase of the review (that is, screening, eligibility and inclusion in meta-analysis)	7

For peer review only

Study records - data collection process	#11c	Describe planned method of extracting data from reports (such as piloting forms, done independently, in duplicate), any processes for obtaining and confirming data from investigators	8
Data items	#12	List and define all variables for which data will be sought (such as PICO items, funding sources), any pre-planned data assumptions and simplifications	8
Outcomes and prioritization	#13	List and define all outcomes for which data will be sought, including prioritization of main and additional outcomes, with rationale	9
Risk of bias in individual studies	#14	Describe anticipated methods for assessing risk of bias of individual studies, including whether this will be done at the outcome or study level, or both; state how this information will be used in data synthesis	9
Data synthesis	#15a	Describe criteria under which study data will be quantitatively synthesised	10
Data synthesis	#15b	If data are appropriate for quantitative synthesis, describe planned summary measures, methods of handling data and methods of combining data from studies, including any planned exploration of consistency (such as I2, Kendall's τ)	10
Data synthesis	#15c	Describe any proposed additional analyses (such as sensitivity or subgroup analyses, meta-regression)	10

For peer review only

Data synthesis	#15d	If quantitative synthesis is not appropriate, describe the type of summary planned	11
Meta-bias(es)	#16	Specify any planned assessment of meta-bias(es) (such as publication bias across studies, selective reporting within studies)	11
Confidence in cumulative evidence	#17	Describe how the strength of the body of evidence will be assessed (such as GRADE)	n/a

The PRISMA-P elaboration and explanation paper is distributed under the terms of the Creative Commons Attribution License CC-BY. This checklist was completed on 17. February 2021 using https://www.goodreports.org/, a tool made by the EQUATOR Network in collaboration with Penelope.ai

For peer review only

BMJ Open

Relationship of prostate cancer topography and tumour conspicuity on multiparametric magnetic resonance imaging: a protocol for a systematic review and meta-analysis

Journal:	*BMJ Open*
Manuscript ID	bmjopen-2021-050376.R1
Article Type:	Protocol
Date Submitted by the Author:	23-Aug-2021
Complete List of Authors:	Satish, Pranav; UCL Medical School, UCL Division of Surgery & Interventional Science<br>Freeman, Alex; University College London Hospitals NHS Foundation Trust, Department of Pathology<br>Kelly, Daniel; Cardiff University, School of Healthcare Sciences<br>Kirkham, Alex; University College London Hospitals NHS Foundation Trust, Department of Radiology<br>Orczyk, Clement; University College London Hospitals NHS Foundation Trust, Department of Urology<br>Simpson, Benjamin Scott; University College London, UCL Cancer Institute<br>Giganti, Francesco; University College London Hospitals NHS Foundation Trust, Department of Radiology<br>Whitaker, Hayley; University College London, Division of Surgery and Interventional Science<br>Emberton, Mark; University College London, Division of Surgery and Interventional Sciences; University College London Hospitals NHS Foundation Trust, Department of Urology<br>Norris, Joseph; University College London, Division of Surgery & Interventional Science
**Primary Subject Heading**:	Urology
Secondary Subject Heading:	Radiology and imaging
Keywords:	Prostate disease < UROLOGY, GENITOURINARY MEDICINE, UROLOGY, Genitourinary imaging < RADIOLOGY & IMAGING

SCHOLARONE™
Manuscripts

Relationship of prostate cancer topography and tumour conspicuity on multiparametric magnetic resonance imaging: a protocol for a systematic review and meta-analysis

Pranav Satish,[1,2] Alex Freeman,[3] Daniel Kelly,[4] Alex Kirkham,[5] Clement Orczyk,[2,6] Benjamin S. Simpson,[7] Francesco Giganti,[2,5] Hayley C. Whitaker,[2] Mark Emberton,[2,6]* Joseph M. Norris,[2,6]*

[1] UCL School of Medicine, University College London, London, UK

[2] UCL Division of Surgery & Interventional Science, University College London, London, UK

[3] Department of Pathology, University College London Hospitals NHS Foundation Trust, London, UK

[4] School of Healthcare Sciences, Cardiff University, Wales, UK

[5] Department of Radiology, University College London Hospitals NHS Foundation Trust, London, UK

[6] Department of Urology, University College London Hospitals NHS Foundation Trust, London, UK

[7] UCL Cancer Institute, University College London, London, UK

** joint-senior authors*

For peer review only

ARTICLE TYPE: Systematic review protocol

WORD COUNT: 2372

KEY WORDS: conspicuity; multiparametric MRI; prostate cancer; systematic review; tumour location

CORRESPONDING AUTHOR: Pranav Satish. UCL Medical School, 74 Huntley Street, Bloomsbury, London, UK, WC1E 6DE. pranav.satish3105@gmail.com.

CONFLICTS OF INTEREST: JMN received funding from the MRC. BSS received funding from the Rosetrees Trust. HCW received funding from PCUK, the Urology Foundation and the Rosetrees Trust. AK, AF and ME have stock interest in Nuada Medical. ME received funding from

NIHR-i4i, MRC, Sonacare, Trod Medical, Cancer Vaccine Institute and Sophiris Biocorp for trials in prostate cancer. ME is a medical consultant to Sonacare, Sophiris Biocorp, Steba Biotech, GSK, Exact Imaging and Profound Medical. Travel allowance was previously provided from Sanofi Aventis, Astellas, GSK and Sonacare. ME is a proctor for HIFU with Sonacare Inc. and paid for training other surgeons in this procedure.

ETHICS: Not required.

FUNDING: Norris is funded by the MRC (MR/S00680X/1).

For peer review only

ABSTRACT

Introduction:

Multiparametric magnetic resonance imaging (mpMRI) has improved the triage of men with suspected prostate cancer, through precision pre-biopsy identification of clinically significant disease. Whilst multiple important characteristics, including tumour grade and size have been shown to affect conspicuity on mpMRI, tumour location and association with mpMRI visibility is an under-explored facet of this field. Therefore, the objective of this systematic review and meta-analysis is to collate the extant evidence comparing MRI performance between different locations within the prostate in men with existing or suspected prostate cancer. This review will help clarify mechanisms that underpin whether a tumour is visible, and the prognostic implications of our findings.

Methods and analysis:

The databases MEDLINE, PubMed, Embase and Cochrane will be systematically searched for relevant studies. Eligible studies will be full-text English-language articles that examine the effect of zonal location on mpMRI conspicuity. Two reviewers will perform study selection, data extraction and quality

assessment. A third reviewer will be involved if consensus is not achieved. The Preferred Reporting Items for Systematic Reviews and Meta-Analyses (PRISMA) guidelines will inform the methodology and reporting of the review. Study bias will be assessed using a modified Newcastle-Ottawa scale. A thematic approach will be used to synthesise key location-based factors associated with mpMRI conspicuity. A meta-analysis will be conducted to form a pooled value of the sensitivity and specificity of mpMRI at different tumour locations.

Ethics and dissemination

Ethical approval is not required as it is a protocol for a systematic review. Findings will be disseminated through peer-reviewed publications and conference presentations.

PROSPERO registration number

CRD42021228087

Strengths and limitations of this study

- This systematic review protocol follows the Preferred Reporting Items for Systematic Review and Meta-Analysis Protocols guidelines.
- This review addresses a gap in the literature with a detailed assessment of the crucial questions surrounding invisible mpMRI lesions
- Due to the limited evidence on the topic the included evidence will be retrospective cohort studies, limiting the strength of conclusions drawn for the thematic synthesis and meta-analysis
- The degree of heterogeneity may limit the generalisability of the results

BACKGROUND

For peer review only

Multiparametric magnetic resonance imaging (mpMRI) has improved the triage of men with suspected prostate cancer, through precision pre-biopsy identification of significant disease. Nevertheless, anywhere from 7% to 55% of prostate cancer lesions may be overlooked by mpMRI (1, 2). As a result, identifying factors that influence visibility of clinically significant cancer has been the subject of extensive research over the past few years (1, 3, 4). Whilst multiple important characteristics, including tumour grade and size have been shown to affect conspicuity on mpMRI (4, 5), tumour location and association with mpMRI visibility remains an under-explored facet of this field.

Although the link between tumour location and conspicuity on mpMRI has not yet been fully elucidated, location does appear to be associated with clinical risk. Transitional zone (TZ) tumours have been associated with lower pathological grade disease (6) and lower rates of biochemical failure following radical prostatectomy (7). There is also increasing evidence to suggest mpMRI-visible lesions may associate with more aggressive clinical features (8). Given this growing body of evidence linking risk, location and conspicuity on mpMRI, the need to amalgamate these features is evident. Therefore, the objective of this systematic review and meta-analysis is to collate the extant evidence regarding the effect of prostate cancer zonal location on mpMRI conspicuity, for the first time. This review will help clarify mechanisms that underpin whether or not a tumour is visible, and the prognostic implications of our findings.

METHODS AND ANALYSIS

This systematic review protocol has been written in line with the Preferred Reporting Items for Systematic Reviews and Meta-Analyses Protocol (PRISMA) 2015 checklist (9). The review was prospectively registered with the PROSPERO review database (CRD42021228087) and will conducted according to the AMSTAR 2 critical appraisal tool (10). All methods described were established prior to the conduct of the review. A systematic review of the PubMed, MEDLINE, Embase and Cochrane databases will be conducted, with the search strategy containing medical subject heading (MeSH) terms for maximum yield of the relevant literature. The search will include MeSH terms for

For peer review only

'prostate cancer' and 'mpMRI,' combined via Boolean operators with synonyms for 'conspicuity' and 'location.' Articles identified by the search strategy will be uploaded to Rayyan (11), a systematic review tool to facilitate the screening process. The included articles' reference section will be screened for further relevant literature not picked up by the initial search strategy.

STUDY SELECTION

The screening process will be conducted by two independent reviewers, removing irrelevant articles based on titles and abstracts. Pertinent studies will be downloaded and their full text assessed to see if they meet the inclusion criteria. Any dispute between reviewers over the relevance of a study will be discussed until concordance is achieved, or a third reviewer will be consulted. The reasons for exclusion will be documented and detailed in the PRISMA flow diasgram. Before starting screening, to minimise inter-reviewer bias, we will conduct calibration exercises to maintain consistency between the two reviewers.

INCLUSION CRITERIA

Studies should compare MRI performance between different locations of the prostate in men with existing or suspected prostate cancer. Zonal location may be defined as traditional McNeal zones (12), biopsy-derived data such as Barzell zones (13), or data acquired via the Ginsburg protocol (14), or any other recognised approach of describing prostate topography. More general definitions of location such as anterior, posterior, base, midgland and apex will also be included. Visibility on mpMRI must be measured by any version of the Prostate Imaging-Reporting and Data System (PI-RADS) (15) or Likert scoring system (16), which is more commonly used in UK-based studies. Other mpMRI scoring schemes (e.g. centre-specific approaches) will not be considered.

EXCLUSION CRITERIA

For peer review only - http://bmjopen.bmj.com/site/about/guidelines.xhtml

Conference abstracts, expert opinions, correspondence articles and case reports will be excluded. Studies that do not correlate zonal location and mpMRI conspicuity will be excluded, as will studies not in the English-language.

DATA EXTRACTION

Like the study selection process, reviewers will then independently extract the relevant study information using the Cochrane data extraction form. Discrepancies between the data extraction will be resolved by consensus. The following study characteristics will be collated: year of publication, authors, study design, patient population, number and age of study participants, mpMRI scoring scheme used (PI-RADS or Likert), definition of clinically significant disease, definition of tumour visibility, sample processing approach, zone(s) assessed and the differential quantification of tumour location. Table 1 summarises data extraction items.

Item	Data Extracted	Data type
1	Author	Study characteristic
2	Publication year	Study characteristic
3	Study design	Study characteristic
4	Patient Population	Demographic
5	Number of patients	Demographic
6	mpMRI protocol	Methodology
7	mpMRI scoring scheme	Methodology
8	Method of pathological correlation	Methodology
9	Definition of clinically significant cancer	Methodology
10	Definition of lesion visibility	Methodology
11	Zones assessed	Outcome
12	Differential quantification of conspicuity	Outcome

Table 1: Data extraction items

For peer review only

ENDPOINTS

The primary endpoint will be the differential tumour visibility on mpMRI, as stratified by location. Secondary endpoints will include explanatory links between location and mpMRI conspicuity, as well as the potential clinical implications. As study methodology may impact the determination of the zone, we will include these variables in a moderator analysis (see Meta-analysis section).

ASSESSMENT OF BIAS

The QUADAS-2 tool to assess bias in diagnostic accuracy studies will be calculated across the included studies (17). The tool assesses risk of bias across 4 domains: patient selection, index test(s), reference standard, flow and timing. Within each section, questions evaluate the quality of the research methodology, at the individual study level, outputting the binary outcome of 'high' or 'low' risk of bias. Both reviewers will independently calculate the score for each study, and any disagreement will be settled by consensus or the involvement of a third reviewer if consensus cannot be achieved. The outcome of the bias assessment will inform the thematic synthesis by providing an assessment of the reliability and applicability of the available evidence. If studies are deemed to be of excessively low quality (or high bias), then these may be excluded from the thematic synthesis at the discretion and consensus of both reviewers. Or, if included, will be accompanied by qualifying commentary in the discussion. For the meta-analysis, studies with 'high' risk of bias in 2 or more domains will be excluded.

META-ANALYSIS

As previously described (18), if there are a sufficient number of studies available that analyse location, using sufficiently comparable methodologies, then we will conduct a meta-analysis. The purpose of the meta-analysis is to obtain a single pooled effect for MRI performance in imaging different areas of the prostate. To limit the effect of methodological heterogeneity on the pooled diagnostic accuracy estimate, we will use two criteria for inclusion in the meta-analysis. Firstly, all studies in the meta-analysis must use multiparametric MRI, rather than single sequence imaging. Secondly, they should define a positive

MRI as a PI-RADS/Likert score of ≥3. Finally, effect estimates for non-randomised and randomised studies will be pooled in separate meta-analyses, as the non-randomised studies will be more affected by bias.

For each topological location reported across studies, the total number of lesions with prostate cancer (m^i), the total number of patients without prostate cancer (n^i) the true positives (y^i, mpMRI visible lesions with confirmed cancer), false positives (z^i, mpMRI visible lesions which are histologically negative), true negatives (m^i-y^i, mpMRI invisible and histologically negative) and false negative (n^i-z^i, mpMRI invisible and present on histological examination) will be recorded. In the case of studies which do not give sufficient detail to derive these values but do list sensitivity or specificity and total patient numbers, these values will be reverse engineered, for example in the case of a study which reports mpMRI sensitivity for a TZ lesion at 0.788 in a group of 60, the true positives can be calculated as $y^i = m^i p^i$ rounded to the nearest integer *or TP = 60 x 0.788 = 47.28* (47.3 true positives). The respective sensitivites and specificities of mpMRI for tumours within differing zonal locations will then be compared. The distribution of untransformed, logit and double arcsine transformed sensitivities and specificities will be compared. Whichever distributions resemble a normal distribution (assessed using density plots and Shapiro-Wilk tests) will be used for further analysis.

The model fitted will be determined based on interstudy variation (measured via I^2); if significant, a random-effect model will be fitted, or a fixed-effect model if not. After fitting a model to all relevant studies, leave-one-out (LOO) analyses and accompanying diagnostic plots will be used to identify influential studies, including externally studentised residuals, difference in fits values, Cook's distances, covariance ratios, LOO estimates of the amount of heterogeneity, LOO values of the test statistics for heterogeneity, hat values and weights. Studies with a statistically significant influence on the fitted model will be removed as outliers and the model refitted. These outliers will be examined for potential confounding variables such as study methodology or poor interobserver agreement for mpMRI scans. Predicted sources of heterogeneity in mpMRI performance include interstudy variation in MRI reader

experience, the MRI scoring system used and the MRI magnet strength used, so these will be accounted for in the interpretation of the results. For each tumour location individual models will first be fit and the pooled sensitivities, specificities, NPV and TPV will be reported. Finally, using tumour location as a subgroup, a bivariate model will be fit to the data and any differences in sensitivity and specificity will be compared using SROC curves and tested using the Chi-squared likelihood-ratio test.

If appropriate, moderator analysis will also be performed between factors, which may influence the outcome, including, but not limited to, study methodology, study size, year of publication, cohort type, relevant methodology (such as radical prostatectomy vs. biopsy studies) or visibility definition. Analytics will be performed as outlined by Wang (18). If there is insufficient data to conduct a meta-analysis, only thematic synthesis will be performed.

DISCUSSION

Following Level 1 evidence support of mpMRI-directed prostate cancer diagnosis (1, 19-21) and implementation into national and international clinical guidelines (22), mpMRI has moved to the forefront of prostate cancer diagnosis. Whilst mpMRI provides refinement of the traditional transrectal ultrasound (TRUS)-guided biopsy approach (1), not all significant prostate cancers are visible on mpMRI, and it is pertinent for us now to explore the reasons that underpin this inconspicuity. Through this systematic review, we will identify the correlation between zonal location and conspicuity, to enhance our understanding of the factors that influence prostate cancer visibility on mpMRI.

Unusual radiological manifestations of prostate cancer are not uncommon due to nuanced histopathological landscape in each zone, rendering MRI signal generation highly varied. In the peripheral zone, several radiological ‘hiding places’ exist, such as very distal apical tumours, subcapsular crescentic tumours, tumours mimicking the posterior midline and infiltrative peripheral zone tumours (23). In the anterior prostate, the presence of benign prostatic hyperplasia (BPH) in the

TZ has been known to generate false positive results, due to difficulty discriminating prostate cancer from BPH nodules (24). Furthermore, MRI signal properties of TZ tumours and normal TZ tissue can be similar (25), further complicating this issue. In the apex of the prostate, the apical capsule is less conspicuous on imaging, and periprostatic fat is usually sparse or completely absent in the apex, which may make the tumor less visible (26). Nevertheless, one study showed high detection rates for this type of tumour (27). Given the many, sometimes contradictory, stances surrounding zonal location and conspicuity in the literature, the planned systematic review will discuss these features, amongst others, in detail.

In summary, this systematic review and meta-analysis will combine the extant evidence in this emerging field, for the first time. Collation and analysis of these data will enrich our understanding of the effect of zonal location on the conspicuity of prostate cancer on mpMRI. Additionally, this process will help reveal the potential clinical role that these effects play in both diagnosis and treatment, thereby aiding identification of important avenues for future research.

TRIAL STATUS

- Preliminary searches: started.
- Piloting of the study selection process: started.
- Formal screening: started
- Data extraction: not started.
- Risk of bias assessment: not started.
- Data analysis: not started.

Draft of search strategy for MEDLINE, EMBASE, PubMed and Cochrane databases

(Prostate cancer OR prostate tumo*r OR prostate lesion)

AND (region* OR position* OR location* OR zone OR zonal location OR midgland OR base OR apex OR AFMS OR anterior OR posterior)

AND (mpMRI OR multiparametric MRI OR multiparametric MR imaging OR mp-mri)

AND (conspicuity OR visibility OR detect* OR visuali* OR identif*)

ETHICS AND DISSEMINATION

Due to the nature of the study, there are no relevant ethical concerns and informed consent will not be required. The protocol and systematic review will be disseminated via a peer-reviewed journal.

REFERENCES

1. Ahmed HU, El-Shater Bosaily A, Brown LC, Gabe R, Kaplan R, Parmar MK, et al. Diagnostic accuracy of multi-parametric MRI and TRUS biopsy in prostate cancer (PROMIS): a paired validating confirmatory study. Lancet. 2017;389(10071):815-22.

2. Johnson DC, Raman SS, Mirak SA, Kwan L, Bajgiran AM, Hsu W, et al. Detection of Individual Prostate Cancer Foci via Multiparametric Magnetic Resonance Imaging. Eur Urol. 2019;75(5):712-20.

3. Norris JM, Carmona Echeverria LM, Bott SRJ, Brown LC, Burns-Cox N, Dudderidge T, et al. What Type of Prostate Cancer Is Systematically Overlooked by Multiparametric Magnetic Resonance Imaging? An Analysis from the PROMIS Cohort. Eur Urol. 2020;78(2):163-70.

4. Huebner NA, Korn S, Resch I, Grubmüller B, Gross T, Gale R, et al. Visibility of significant prostate cancer on multiparametric magnetic resonance imaging (MRI)-do we still need contrast media? Eur Radiol. 2021;31(6):3754-64.

5. Bratan F, Niaf E, Melodelima C, Chesnais AL, Souchon R, Mège-Lechevallier F, et al. Influence of imaging and histological factors on prostate cancer detection and localisation on multiparametric MRI: a prospective study. Eur Radiol. 2013;23(7):2019-29.

6. Lee JJ, Thomas IC, Nolley R, Ferrari M, Brooks JD, Leppert JT. Biologic differences between peripheral and transition zone prostate cancer. Prostate. 2015;75(2):183-90.

For peer review only

7. Shin N, Park SY. Postoperative Biochemical Failure in Patients With PI-RADS Category 4 or 5 Prostate Cancers: Risk Stratification According to Zonal Location of an Index Lesion. AJR Am J Roentgenol. 2020;215(4):913-9.

8. Norris JM, Simpson BS, Freeman A, Kirkham A, Whitaker HC, Emberton M. Conspicuity of prostate cancer on multiparametric magnetic resonance imaging: A cross-disciplinary translational hypothesis. Faseb j. 2020;34(11):14150-9.

9. Shamseer L, Moher D, Clarke M, Ghersi D, Liberati A, Petticrew M, et al. Preferred reporting items for systematic review and meta-analysis protocols (PRISMA-P) 2015: elaboration and explanation. Bmj. 2015;350:g7647.

10. Shea BJ, Reeves BC, Wells G, Thuku M, Hamel C, Moran J, et al. AMSTAR 2: a critical appraisal tool for systematic reviews that include randomised or non-randomised studies of healthcare interventions, or both. Bmj. 2017;358:j4008.

11. Ouzzani M, Hammady H, Fedorowicz Z, Elmagarmid A. Rayyan-a web and mobile app for systematic reviews. Syst Rev. 2016;5(1):210.

12. McNeal JE. The zonal anatomy of the prostate. Prostate. 1981;2(1):35-49.

13. Valerio M, Anele C, Charman SC, van der Meulen J, Freeman A, Jameson C, et al. Transperineal template prostate-mapping biopsies: an evaluation of different protocols in the detection of clinically significant prostate cancer. BJU Int. 2016;118(3):384-90.

14. Hansen N, Patruno G, Wadhwa K, Gaziev G, Miano R, Barrett T, et al. Magnetic Resonance and Ultrasound Image Fusion Supported Transperineal Prostate Biopsy Using the Ginsburg Protocol: Technique, Learning Points, and Biopsy Results. Eur Urol. 2016;70(2):332-40.
15. Turkbey B, Rosenkrantz AB, Haider MA, Padhani AR, Villeirs G, Macura KJ, et al. Prostate Imaging Reporting and Data System Version 2.1: 2019 Update of Prostate Imaging Reporting and Data System Version 2. Eur Urol. 2019;76(3):340-51.

16. Norris JM, Simpson BS, Ball R, Freeman A, Kirkham A, Parry MA, et al. A Modified Newcastle-Ottawa Scale for Assessment of Study Quality in Genetic Urological Research. Eur Urol. 2021;79(3):325-6.

17. Whiting PF, Rutjes AW, Westwood ME, Mallett S, Deeks JJ, Reitsma JB, et al. QUADAS-2: a revised tool for the quality assessment of diagnostic accuracy studies. Ann Intern Med. 2011;155(8):529-36.

18. Wang N. How to Conduct a Meta-Analysis of Proportions in R: A Comprehensive Tutorial2018.

19. Kasivisvanathan V, Rannikko AS, Borghi M, Panebianco V, Mynderse LA, Vaarala MH, et al. MRI-Targeted or Standard Biopsy for Prostate-Cancer Diagnosis. N Engl J Med. 2018;378(19):1767-77.

For peer review only

20. Simmons LAM, Kanthabalan A, Arya M, Briggs T, Barratt D, Charman SC, et al. The PICTURE study: diagnostic accuracy of multiparametric MRI in men requiring a repeat prostate biopsy. Br J Cancer. 2017;116(9):1159-65.

21. Rouvière O, Puech P, Renard-Penna R, Claudon M, Roy C, Mège-Lechevallier F, et al. Use of prostate systematic and targeted biopsy on the basis of multiparametric MRI in biopsy-naive patients (MRI-FIRST): a prospective, multicentre, paired diagnostic study. Lancet Oncol. 2019;20(1):100-9.

22. Wise J. NICE recommends MRI for suspected prostate cancer to reduce biopsies. Bmj. 2018;363:k5290.

23. Rosenkrantz AB, Verma S, Turkbey B. Prostate cancer: top places where tumors hide on multiparametric MRI. AJR Am J Roentgenol. 2015;204(4):W449-56.

24. Hoeks CM, Hambrock T, Yakar D, Hulsbergen-van de Kaa CA, Feuth T, Witjes JA, et al. Transition zone prostate cancer: detection and localization with 3-T multiparametric MR imaging. Radiology. 2013;266(1):207-17.

25. Akin O, Sala E, Moskowitz CS, Kuroiwa K, Ishill NM, Pucar D, et al. Transition zone prostate cancers: features, detection, localization, and staging at endorectal MR imaging. Radiology. 2006;239(3):784-92.

26. Tan N, Margolis DJ, Lu DY, King KG, Huang J, Reiter RE, et al. Characteristics of Detected and Missed Prostate Cancer Foci on 3-T Multiparametric MRI Using an Endorectal Coil Correlated With Whole-Mount Thin-Section Histopathology. AJR Am J Roentgenol. 2015;205(1):W87-92.

27. Nix JW, Turkbey B, Hoang A, Volkin D, Yerram N, Chua C, et al. Very distal apical prostate tumours: identification on multiparametric MRI at 3 Tesla. BJU Int. 2012;110(11 Pt B):E694-700.

For peer review only

FOOTNOTES

Twitter: @PranavSatish7, @MrJosephNorris, @b3nsimp, @HayleyWhitaker, @EmbertonMark, @profdkelly, @giga_fra

Contributors: The authors' contribution includes, but is not limited to, the following: PS and JMN drafted the manuscript and created the study concept. BS provided the meta-analysis section. FG, AF, DK, AK, CO, HCW and ME provided supervision and guidance. All authors reviewed and approved the manuscript in its current form.

Funding: JMN is funded by the Medical Research Council (MRC) on an MRC Clinical Research Training Fellowship (MRC Grant Ref: MR/S00680X/1). BSS receives funding from the Rosetrees Foundation.

Competing interests: JMN received funding from the MRC. BSSS received funding from the Rosetrees Trust. HCW received funding from PCUK, the Urology Foundation and the Rosetrees Trust. AK, AF and ME have stock interest in Nuada Medical. ME received funding from NIHR-i4i, MRC, Sonacare, Trod Medical, Cancer Vaccine Institute and Sophiris Biocorp for trials in prostate cancer. ME is a medical consultant to Sonacare, Sophiris Biocorp, Steba Biotech, GSK, Exact Imaging and Profound Medical. Travel allowance was previously provided from Sanofi Aventis, Astellas, GSK and Sonacare. ME is a proctor for HIFU with Sonacare Inc. and paid for training other surgeons in this procedure.

Patient and public involvement; Patients and/or the public were not involved in the design, conduct, reporting or dissemination plans of this research.

Patient consent for publication: Not required.

Provenance and peer review: Not commissioned; externally peer reviewed.

For peer review only

Reporting checklist for protocol of a systematic review and meta analysis.

Based on the PRISMA-P guidelines.

Instructions to authors

Complete this checklist by entering the page numbers from your manuscript where readers will find each of the items listed below.

Your article may not currently address all the items on the checklist. Please modify your text to include the missing information. If you are certain that an item does not apply, please write "n/a" and provide a short explanation.

Upload your completed checklist as an extra file when you submit to a journal.

In your methods section, say that you used the PRISMA-Preporting guidelines, and cite them as:

Moher D, Shamseer L, Clarke M, Ghersi D, Liberati A, Petticrew M, Shekelle P, Stewart LA. Preferred Reporting Items for Systematic Review and Meta-Analysis Protocols (PRISMA-P) 2015 statement. Syst Rev. 2015;4(1):1.

		Reporting Item	Page Number
**Title**			
Identification	#1a	Identify the report as a protocol of a systematic review	7
Update	#1b	If the protocol is for an update of a previous systematic review, identify as such	n/a

For peer review only

**Registration**			
	#2	If registered, provide the name of the registry (such as PROSPERO) and registration number	5
**Authors**			
Contact	#3a	Provide name, institutional affiliation, e-mail address of all protocol authors; provide physical mailing address of corresponding author	3
Contribution	#3b	Describe contributions of protocol authors and identify the guarantor of the review	18
**Amendments**			
	#4	If the protocol represents an amendment of a previously completed or published protocol, identify as such and list changes; otherwise, state plan for documenting important protocol amendments	n/a
**Support**			
Sources	#5a	Indicate sources of financial or other support for the review	3
Sponsor	#5b	Provide name for the review funder and / or sponsor	n/a
Role of sponsor or funder	#5c	Describe roles of funder(s), sponsor(s), and / or institution(s), if any, in developing the protocol	n/a
**Introduction**			

For peer review only

Rationale	#6	Describe the rationale for the review in the context of what is already known	6
Objectives	#7	Provide an explicit statement of the question(s) the review will address with reference to participants, interventions, comparators, and outcomes (PICO)	7
**Methods**			
Eligibility criteria	#8	Specify the study characteristics (such as PICO, study design, setting, time frame) and report characteristics (such as years considered, language, publication status) to be used as criteria for eligibility for the review	8
Information sources	#9	Describe all intended information sources (such as electronic databases, contact with study authors, trial registers or other grey literature sources) with planned dates of coverage	7
Search strategy	#10	Present draft of search strategy to be used for at least one electronic database, including planned limits, such that it could be repeated	13
Study records - data management	#11a	Describe the mechanism(s) that will be used to manage records and data throughout the review	7
Study records - selection process	#11b	State the process that will be used for selecting studies (such as two independent reviewers) through each phase of the review (that is, screening, eligibility and inclusion in meta-analysis)	7

For peer review only

Study records - data collection process	#11c	Describe planned method of extracting data from reports (such as piloting forms, done independently, in duplicate), any processes for obtaining and confirming data from investigators	8
Data items	#12	List and define all variables for which data will be sought (such as PICO items, funding sources), any pre-planned data assumptions and simplifications	8
Outcomes and prioritization	#13	List and define all outcomes for which data will be sought, including prioritization of main and additional outcomes, with rationale	9
Risk of bias in individual studies	#14	Describe anticipated methods for assessing risk of bias of individual studies, including whether this will be done at the outcome or study level, or both; state how this information will be used in data synthesis	9
Data synthesis	#15a	Describe criteria under which study data will be quantitatively synthesised	10
Data synthesis	#15b	If data are appropriate for quantitative synthesis, describe planned summary measures, methods of handling data and methods of combining data from studies, including any planned exploration of consistency (such as I2, Kendall's τ)	10
Data synthesis	#15c	Describe any proposed additional analyses (such as sensitivity or subgroup analyses, meta-regression)	10

For peer review only

Data synthesis	#15d	If quantitative synthesis is not appropriate, describe the type of summary planned	11
Meta-bias(es)	#16	Specify any planned assessment of meta-bias(es) (such as publication bias across studies, selective reporting within studies)	11
Confidence in cumulative evidence	#17	Describe how the strength of the body of evidence will be assessed (such as GRADE)	n/a

The PRISMA-P elaboration and explanation paper is distributed under the terms of the Creative Commons Attribution License CC-BY. This checklist was completed on 17. February 2021 using https://www.goodreports.org/, a tool made by the EQUATOR Network in collaboration with Penelope.ai

For peer review only
